# Trends in Severe Outcomes Among Adult and Pediatric Patients Hospitalized With COVID-19 in the Canadian Nosocomial Infection Surveillance Program, March 2020 to May 2022

**DOI:** 10.1001/jamanetworkopen.2023.9050

**Published:** 2023-04-20

**Authors:** Robyn Mitchell, Joelle Cayen, Nisha Thampi, Charles Frenette, Jessica Bartoskzo, Kelly Baekyung Choi, Jeannette L. Comeau, John Conly, Chelsey Ellis, Jennifer Ellison, John Embil, Gerald Evans, Lynn Johnston, Jennie Johnstone, Kevin C. Katz, Pamela Kibsey, Bonita Lee, Marie-Astrid Lefebvre, Yves Longtin, Allison McGeer, Dominik Mertz, Jessica Minion, Wallis Rudnick, Anada Silva, Stephanie W. Smith, Jocelyn A. Srigley, Kathryn N. Suh, Jen Tomlinson, Alice Wong, Linda Pelude

**Affiliations:** 1 Centre for Communicable Diseases and Infection Control, Public Health Agency of Canada, Ottawa, Ontario, Canada; 2 Department of Pediatrics, Children’s Hospital of Eastern Ontario, Ottawa, Ontario, Canada; 3 Division of Infectious Diseases, Department of Medicine, McGill University Health Centre, Montréal, Québec, Canada; 4 Department of Pediatrics, Dalhousie University, Halifax, Nova Scotia, Canada; 5 Department of Medicine, University of Calgary, Calgary, Alberta, Canada; 6 Department of Laboratory Medicine, The Moncton Hospital, Moncton, New Brunswick, Canada; 7 Infection, Prevention and Control, Alberta Health Services, Calgary, Alberta, Canada; 8 Infection Prevention and Control, Health Sciences Centre, Winnipeg, Manitoba, Canada; 9 Division of Infectious Diseases, Queen’s University, Kingston, Ontario, Canada; 10 Division of Infectious Diseases, Department of Medicine, Queen Elizabeth II Health Sciences Centre, Halifax, Nova Scotia, Canada; 11 Infection Prevention and Control, Sinai Health, Toronto, Ontario, Canada; 12 Infection Prevention and Control, North York General Hospital, Toronto, Ontario, Canada; 13 Department of Pathology and Laboratory Medicine, Royal Jubilee Hospital, Victoria, British Columbia, Canada; 14 Department of Pediatrics, Stollery Children’s Hospital, Edmonton, Alberta, Canada; 15 Montreal Children’s Hospital, McGill University Health Centre, Montréal, Québec, Canada; 16 Infection Prevention and Control, SMBD Jewish General Hospital, Montréal, Québec, Canada; 17 Division of Infectious Diseases, Department of Medicine, McMaster University and Hamilton Health Sciences, Hamilton, Ontario, Canada; 18 Department of Laboratory Medicine, Saskatchewan Health Authority, Regina, Saskatchewan, Canada; 19 Faculty of Medicine, University of Alberta Hospital, Edmonton, Alberta, Canada; 20 Infection Prevention and Control, BC Women’s and BC Children’s Hospital, Vancouver, British Columbia, Canada; 21 Infection Prevention and Control, The Ottawa Hospital, Ottawa, Ontario, Canada; 22 Division of Infectious Diseases, Department of Medicine, Royal University Hospital, Saskatoon, Saskatchewan, Canada

## Abstract

**Importance:**

Trends in COVID-19 severe outcomes have significant implications for the health care system and are key to informing public health measures. However, data summarizing trends in severe outcomes among patients hospitalized with COVID-19 in Canada are not well described.

**Objective:**

To describe trends in severe outcomes among patients hospitalized with COVID-19 during the first 2 years of the COVID-19 pandemic.

**Design, Setting, and Participants:**

Active prospective surveillance in this cohort study was conducted from March 15, 2020, to May 28, 2022, at a sentinel network of 155 acute care hospitals across Canada. Participants included adult (aged ≥18 years) and pediatric (aged 0-17 years) patients hospitalized with laboratory-confirmed COVID-19 at a Canadian Nosocomial Infection Surveillance Program (CNISP)–participating hospital.

**Exposures:**

COVID-19 waves, COVID-19 vaccination status, and age group.

**Main Outcomes and Measures:**

The CNISP collected weekly aggregate data on the following severe outcomes: hospitalization, admission to an intensive care unit (ICU), receipt of mechanical ventilation, receipt of extracorporeal membrane oxygenation, and all-cause in-hospital death.

**Results:**

Among 1 513 065 admissions, the proportion of adult (n = 51 679) and pediatric (n = 4035) patients hospitalized with laboratory-confirmed COVID-19 was highest in waves 5 and 6 of the pandemic compared with waves 1 to 4 (77.3 vs 24.7 per 1000 patient admissions). Despite this, the proportion of patients with positive test results for COVID-19 who were admitted to an ICU, received mechanical ventilation, received extracorporeal membrane oxygenation, and died were each significantly lower in waves 5 and 6 when compared with waves 1 through 4. Admission to the ICU and in-hospital all-cause death rates were significantly higher among those who were unvaccinated against COVID-19 when compared with those who were fully vaccinated (incidence rate ratio, 4.3 and 3.9, respectively) or fully vaccinated with an additional dose (incidence rate ratio, 12.2 and 15.1, respectively).

**Conclusions and Relevance:**

The findings of this cohort study of patients hospitalized with laboratory-confirmed COVID-19 suggest that COVID-19 vaccination is important to reduce the burden on the Canadian health care system as well as severe outcomes associated with COVID-19.

## Key Points

**Question:**

What are the trends in severe outcomes among adult and pediatric patients hospitalized with laboratory-confirmed COVID-19 during the first 2 years of the COVID-19 pandemic?

**Findings:**

In a cohort study of 55 714 adult and pediatric patients hospitalized with laboratory-confirmed COVID-19 in 155 acute care hospitals in Canada, surveillance data show that during waves 5 and 6 of the COVID-19 pandemic, hospitals experienced a surge in patients hospitalized with COVID-19, an increase in nosocomial transmission of COVID-19, and a decrease in severe outcomes.

**Meaning:**

These findings suggest that despite a reduction in severe outcomes in waves 5 and 6, the burden of COVID-19 on Canadian hospitals was substantial.

## Teaser

This cohort study describes trends in severe outcomes among patients hospitalized with COVID-19 from March 2020 to May 2022 in the Canadian Nosocomial Infection Surveillance Program.

## Introduction

The clinical presentation of COVID-19 can range from asymptomatic infection to mild, moderate, severe, or critical disease. Severe and critical disease occur more often in older persons and/or those with preexisting comorbidities^1^ as well as those who lack immunity either through vaccination or prior infection.^2,3,4,5^ Changes in severe outcomes have significant implications for the health care system. Therefore, it is important to monitor trends in severe outcomes associated with COVID-19 to inform public health measures.

Data summarizing trends in severe outcomes among patients hospitalized with COVID-19 in Canada are limited. Using data from a large network of hospitals, we describe trends in severe outcomes among patients hospitalized with COVID-19 from March 2020 to May 2022.

## Methods

This study was considered either exempt from the requirement for ethics approval and informed consent within the mandate of hospital infection prevention and control programs or approved by the research ethics boards at participating hospitals if required by institution-specific policies. This study followed the Strengthening the Reporting of Observational Studies in Epidemiology (STROBE) reporting guideline.

### Data Source and Data Collection

The Canadian Nosocomial Infection Surveillance Program (CNISP) is a collaboration between the Public Health Agency of Canada, the Association of Medical Microbiology and Infectious Disease Canada, and sentinel hospitals. Since March 2020, CNISP has collected weekly aggregate data (stratified by age group, vaccination status, and source of acquisition) on (1) patients hospitalized with COVID-19, (2) patients with a positive test result for COVID-19 (hereinafter referred to as COVID-19–positive patients) admitted to an intensive care unit (ICU), (3) COVID-19–positive patients receiving mechanical ventilation, (4) COVID-19–positive patients receiving extracorporeal membrane oxygenation (ECMO), and (5) all-cause in-hospital death. Hospitalization with COVID-19 included patients who were admitted due to COVID-19 and COVID-19–positive patients whose admission was unrelated to COVID-19 (eg, incidental admission or health care acquired). Data were collected through review of patient medical records by trained infection control professionals at 155 acute care hospitals from 10 provinces and 1 territory, representing approximately 31% of all Canadian acute care beds. This study includes patients hospitalized with laboratory-confirmed COVID-19 between March 15, 2020, and May 28, 2022.

### Definitions

Patients of any age whose first positive test result for SARS-CoV-2 using reverse transcription–polymerase chain reaction was within 14 days prior to admission or while in hospital were eligible for inclusion. A health care–associated case was defined as (1) symptom onset or positive test result at least 7 calendar days after admission to hospital, (2) readmission with a positive test result within 7 days after discharge from hospital, or (3) best clinical judgment (eg, symptom onset <7 days but known epidemiological link to a positive inpatient or staff case).

Based on the weeks when we observed an increase in the proportion of COVID-19 hospitalizations in our network, we identified time periods for the study. These included wave 1 (March 15 to August 31, 2020 [wild-type variant dominant]), wave 2 (September 1, 2020, to February 28, 2021 [wild-type variant dominant]), wave 3 (March 1 to June 30, 2021 [mixed Alpha, Beta, and/or Gamma variants]), wave 4 (July 1 to December 25, 2021 [Alpha variant dominant]), wave 5 (December 26, 2021, to March 19, 2022 [Omicron variant dominant]), and wave 6 (March 20 to May 28, 2022 [Omicron variant dominant]).

Fully vaccinated was defined as a patient with symptom onset or specimen collection date that was 14 or more days following receipt of a second dose of a 2-dose COVID-19 vaccine or a single dose of a 1-dose COVID-19 vaccine. Fully vaccinated with an additional dose was defined as a fully vaccinated patient with symptom onset or specimen collection date that was 14 or more days following receipt of at least 1 additional dose of a COVID-19 vaccine.

### Statistical Analysis

The weekly proportion of severe outcomes was calculated using the number of new adult (aged ≥18 years) and pediatric (aged 0-17 years) COVID-19–positive patients per 1000 patient admissions. Severe outcomes were defined as hospitalization, admission to an ICU, receipt of mechanical ventilation, receipt of ECMO, and all-cause in-hospital death (defined as death during the hospital stay). Weekly patient admissions were estimated by dividing quarterly patient admissions in 2020 and 2021 by the number of weeks in each quarter. There were no significant differences in the proportions of severe outcomes between waves 1 through 4 inclusive and waves 5 and 6, with the exception of a significant difference in the proportion of all-cause in-hospital deaths among adult patients between waves 1 and 2 and waves 3 and 4. Further, our primary comparison of interest was waves 5 and 6 compared with earlier waves. Because of this statistical and clinical rationale, we opted to pool adult and pediatric data from waves 1 through 4 inclusive and waves 5 and 6 together for all severe outcomes. For all-cause in-hospital mortality among adult patients, we pooled waves 1 and 2, waves 3 and 4, and waves 5 and 6 together. Unadjusted odd ratios (OR) and 95% CIs were calculated to compare the proportion of severe outcomes between pooled waves.

The proportion of patients who acquired COVID-19 in the hospital was compared between waves 2 through 4 inclusive and waves 5 and 6. Aggregate data on acquisition status were not collected during the first wave. Differences in proportions were compared using the χ^2^ test. Two-tailed *P* ≤ .05 was considered statistically significant.

Cumulative incidence rates by COVID-19 vaccination status for ICU admission and all-cause in-hospital death were calculated for patients hospitalized with COVID-19 during waves 5 and 6, where vaccination status data were available. Age-standardized incidence rate ratios (IRRs) and 95% CIs were calculated to compare these rates between vaccination status groups. Population vaccine coverage data to May 22, 2022, were obtained from the Public Health Agency of Canada’s COVID-19 vaccination coverage data.^6^ The denominator was adjusted to take into account that the CNISP captures approximately one-third of Canadian patient admissions. Estimates were age-standardized using 2016 Statistics Canada population estimates.^7^ Patients aged 0 to 4 years were excluded from IRR calculations as COVID-19 vaccines were not available for this age group in waves 5 and 6. Patients from Alberta, Yukon, and the Northwest Territories were excluded from IRR calculations as vaccination status data were not available. All analyses were conducted using R, version 4.1.2 (R Project for Statistical Computing).

## Results

**Figure 1.**
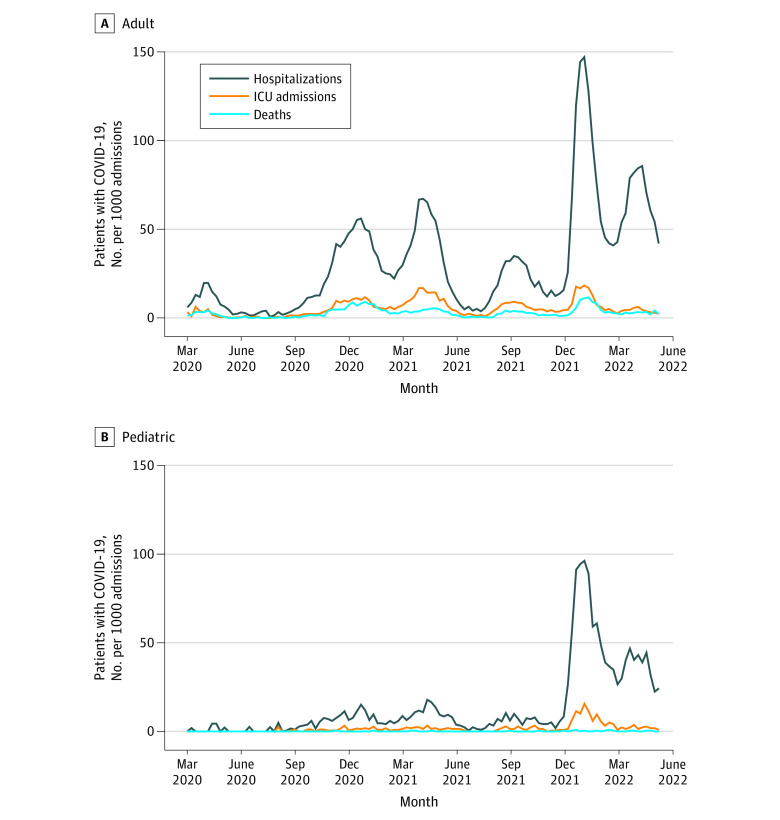
Weekly Proportion of Adult and Pediatric Patients Hospitalized With COVID−19, Patients Admitted to an Intensive Care Unit (ICU), and All-Cause In-Hospital Deaths per 1000 Patient Admissions Events occurred from March 15, 2020, to May 28, 2022. Data were obtained from 64 adult, mixed, and pediatric Canadian Nosocomial Infection Surveillance Program hospitals with age-stratified denominator data. Adults were 18 years or older; pediatric patients, younger than 18 years.

From March 15, 2020, to May 28, 2022, 155 hospitals with 1 513 065 admissions reported a total of 51 679 adult and 4035 pediatric patients hospitalized with laboratory-confirmed COVID-19, with 8683 adults (16.8%) and 498 children (12.3%) admitted to an ICU. Among all patient admissions, the proportion of COVID-19 hospitalizations (adult and pediatric patients) for waves 1 through 4 combined was 24.7 per 1000 patient admissions compared with 77.3 per 1000 patient admissions for waves 5 and 6. Among all patient admissions, the proportion of COVID-19–positive patients admitted to an ICU during waves 1 through 4 combined was 5.3 per 1000 patient admissions compared with 6.8 per 1000 patient admissions for waves 5 and 6 (Table). Figure 1 presents the proportion of adult and pediatric patients hospitalized with COVID-19, admitted to an ICU, and who died in the hospital (all-cause), among those hospitals where age-stratified denominator data were available. The weekly proportion of adult and pediatric patients hospitalized with COVID-19 peaked at 146.8 and 96.3 per 1000 patient admissions, respectively, during the week of January 16, 2022 (wave 5), surpassing the peaks of all previous and subsequent waves. The highest proportion of adult ICU COVID-19 admissions was 18.3 per 1000 patient admissions and the highest proportion of pediatric ICU COVID-19 admissions was 15.6 per 1000 patient admissions during the week of January 16, 2022 (wave 5). Among 51 496 patients hospitalized with COVID-19 for whom acquisition was known, 7012 (13.6%) acquired COVID-19 in the hospital. A significantly higher proportion of patients acquired COVID-19 in the hospital in waves 5 and 6 (combined 4051 of 23 973 [16.9%]) compared with waves 2 through 4 (combined 2961 of 27 523 [10.8%]; *P* < .001).

**Table.** Adult and Pediatric Patients Who Were Hospitalized With COVID-19, Admitted to an ICU, and Died in the Hospital^a^

Outcome	Wave 1	Wave 2	Wave 3	Wave 4	Wave 5	Wave 6
Total No. of patients hospitalized with COVID-19	851	10 992	8531	9568	14 461	9073
Total No. of COVID-19–positive patients admitted to ICU	173	2068	2057	2164	1551	520
Total No. of COVID-19–positive patients who died (all-cause)	144	1663	714	1095	1074	398
Total No. of patient admissions	173 177	390 156	257 646	389 338	167 327	135 421

Abbreviation: ICU, intensive care unit.

^a^ Includes adult and pediatric patients in 155 hospitals.

**Figure 2.**
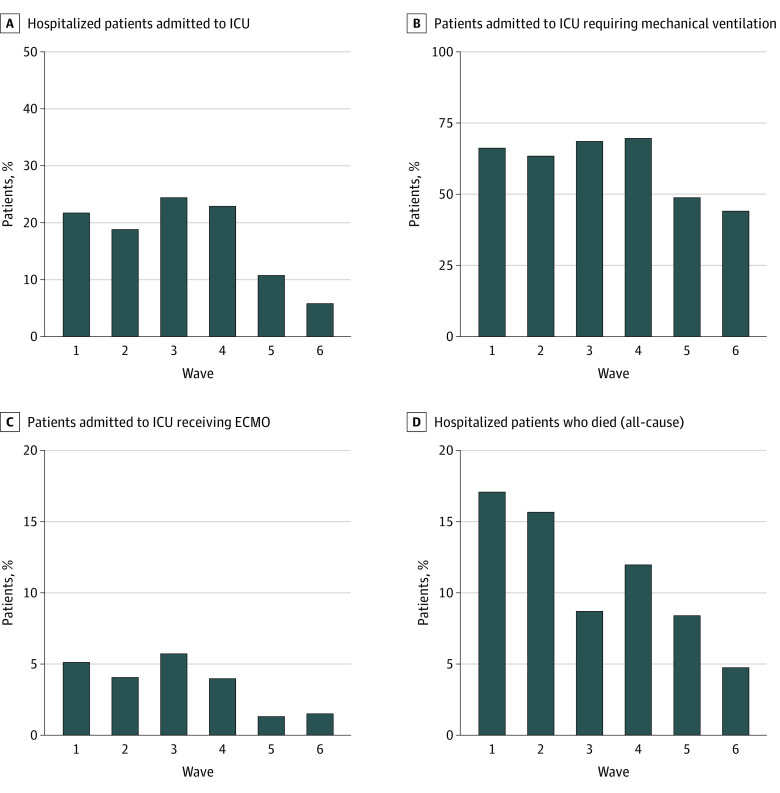
Proportion of Severe Outcomes Among Adult Patients Hospitalized With COVID-19 by Wave Data are from 153 hospitals. ECMO indicates extracorporeal membrane oxygenation; ICU, intensive care unit.

Figure 2 presents the proportion of severe outcomes among adult patients hospitalized with COVID-19 by wave. The proportion of adult patients hospitalized with COVID-19 who were admitted to an ICU was significantly lower in waves 5 and 6 (combined 1819 of 20 858 [8.7%]) when compared with waves 1 through 4 (combined 6710 of 30 821 [21.8%]; OR, 0.34 [95% CI, 0.32-0.36]). Among adult patients in the ICU, the proportion who received mechanical ventilation during waves 5 and 6 (combined 866 of 1819 [47.6%]) was significantly lower when compared with waves 1 through 4 (combined 4507 of 6710 [67.2%]; OR, 0.44 [95% CI, 0.40-0.49]). The proportion of adult patients in the ICU who received ECMO was significantly lower during waves 5 and 6 (combined 24 of 1819 [1.3%]) when compared with waves 1 through 4 (combined 310 of 6710 [4.6%]; OR, 0.28 [95% CI, 0.18-0.41]). The proportion of adult patients hospitalized with COVID-19 who died in the hospital (all-cause) significantly decreased from waves 1 and 2 (2175 of 13 601 [16.0%]) to waves 3 and 4 (1799 of 17 220 [10.4%]) to waves 5 and 6 (1453 of 20 858 [7.0%]) (*P* < .001).

**Figure 3.**
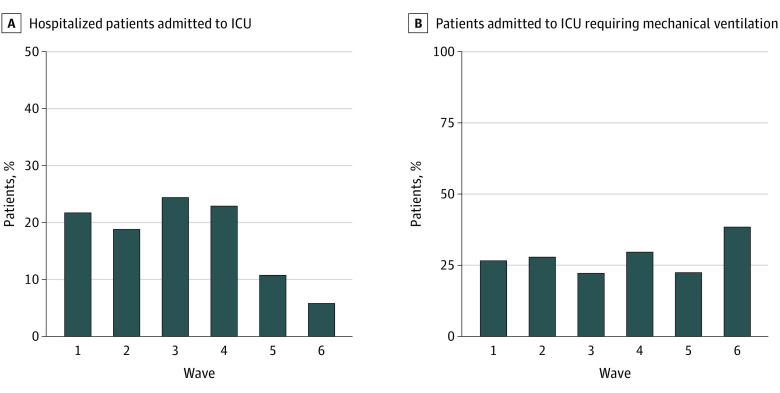
Proportion of Severe Outcomes Among Pediatric Patients Hospitalized With COVID-19 by Wave Data are from 81 hospitals. ICU indicates intensive care unit.

Figure 3 presents the proportion of severe outcomes among pediatric patients hospitalized with COVID-19 by wave. The proportion of pediatric patients hospitalized with COVID-19 who were admitted to an ICU was significantly lower in waves 5 and 6 (combined 252 of 2676 [9.4%]) when compared with waves 1 through 4 (combined 246 of 1359 [18.1%]; OR, 0.47 [95% CI, 0.39-0.57]). Among pediatric patients in the ICU, the proportion who received mechanical ventilation during waves 5 and 6 (combined 65 of 252 [25.8%]) was similar when compared with waves 1 through 4 (combined 66 of 246 [26.8%]; OR, 0.95 [95% CI, 0.64-1.41]). Overall, 1 pediatric patient hospitalized with COVID-19 received ECMO. Thirty-one deaths were reported among pediatric patients hospitalized with COVID-19. Twelve all-cause in-hospital deaths among 1359 pediatric patients were reported in waves 1 through 4 combined (0.9%), which was similar to waves 5 and 6 combined (19 of 2676 [0.7%]), although this finding was not significant (*P* = .60).

**Figure 4.**
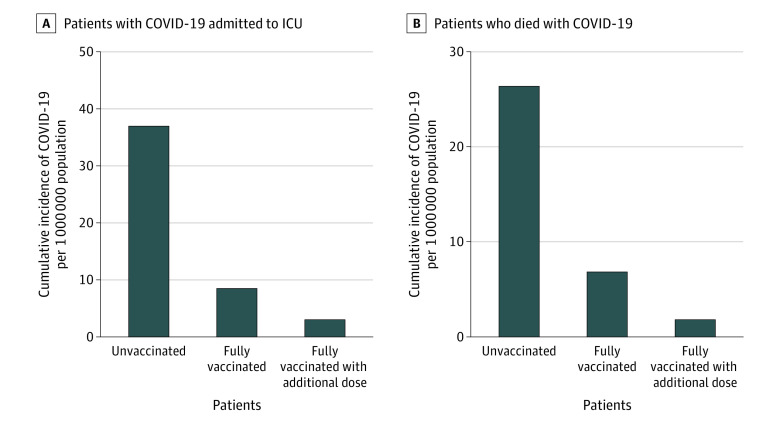
Cumulative Age-Standardized Incident Rates of COVID-19–Positive Patients Admitted to the Intensive Care Unit (ICU) and Who Died (In-Hospital, All Causes) Data are stratified by vaccination status per 1 000 000 population and from 58 hospitals in waves 5 to 6. Denominators were adjusted to reflect that approximately one-third of hospitalized patients in Canada are captured by the Canadian Nosocomial Infection Surveillance Program. Partially vaccinated patients (defined as patients with a symptom onset or specimen collection date that was ≥14 days following receipt of a first dose of a 2-dose COVID-19 vaccine or <14 days after receiving a second dose) were excluded from these analyses. Vaccine coverage data are from the Public Health Agency of Canada.

During waves 5 and 6, the age-standardized incidence rate for ICU admission among unvaccinated patients was 4.3 times higher than in fully vaccinated patients (95% CI, 2.1-9.6) and 12.2 times higher than in fully vaccinated patients with at least 1 additional dose (95% CI, 4.4-50.1). The age-standardized incidence rate for all-cause in-hospital death among unvaccinated patients vs fully vaccinated patients was 3.9 (95% CI, 1.8-9.9) and among unvaccinated patients vs fully vaccinated with at least 1 additional dose was 15.1 (95% CI, 4.3-110.0) (Figure 4).

## Discussion

Our findings from a large sentinel network of Canadian hospitals indicate that although the proportion of patients hospitalized with COVID-19 was highest in wave 5, a significantly smaller proportion of hospitalized adult and pediatric patients with COVID-19 were admitted to an ICU, and a significantly smaller proportion of adult patients with COVID-19 received mechanical ventilation or ECMO or died during later waves vs earlier waves. These findings are likely due to multiple factors, including higher COVID-19 vaccination coverage during later waves,^2,6,8,9,10^ the emergence of a potentially less virulent SARS-CoV-2 variant,^11,12,13^ prior natural immunity,^4^ and improvements in COVID-19 management over time.^14,15,16^ Another potential factor is the inclusion of incidental admissions in our case definition; the inclusion of COVID-19–positive patients admitted for reasons other than COVID-19 may underestimate the proportion of severe outcomes, particularly during waves 5 and 6. Nonetheless, our results are consistent with evidence from other studies^3,9,17,18,19,20^ that have also found decreases in severe outcomes among patients hospitalized with COVID-19 during a similar surveillance period.

Our findings show a significant increase in acquisition of COVID-19 among patients within Canadian hospitals during waves 5 and 6. Reasons for this are uncertain; however, factors that may have increased opportunities for hospital transmission include a high incidence of COVID-19 in the community with subsequent admission of larger numbers of patients with incidental infection that was initially missed; an increase in community-acquired COVID-19 among health care workers and visitors, leading to an increase in patient exposure; increase in transmissibility of Omicron variants in waves 5 and 6; a potential decrease in adherence to infection control practices by patients, visitors, and staff as the perceived risk of COVID-19 waned; personal protective equipment fatigue; and the discontinuation of public health measures in the community. Our findings are similar to those of a report from England^21^ in which 20.3% of patients hospitalized with COVID-19 were first identified more than 7 days after hospital admission in December 2021 compared with only 8.3% of patients in early November 2021.

During waves 5 and 6, the cumulative incident rates of ICU admission and all-cause in-hospital deaths were significantly higher for unvaccinated patients compared with fully vaccinated patients and patients fully vaccinated with an additional dose. This is consistent with findings of studies from the US^22,23^ showing that ICU and mortality rates were highest among unvaccinated patients and lowest among those who had received an additional dose.

### Limitations

This study has several limitations. Our analyses did not take into account immunity due to previous infection or other factors such as improved treatments, which may contribute to the observed reduction in severe outcomes. Patient admissions were used to calculate the proportion of all admissions, as reliable population denominator estimates were not available for the catchment areas of the hospitals included in our study. Although we observed a decrease in patient admissions in wave 1 (eg, suspension of elective procedures), patient admissions were comparable in waves 2 through 6 and provide a standardized denominator for the comparison of trends. Our data include incidental COVID-19 hospitalizations, which may underestimate the proportion of severe outcomes among patients in hospitals included in this cohort during waves 5 and 6. Our findings may also underestimate the proportion of health care–associated cases, given the shorter incubation period for variants circulating during waves 5 and 6; however, medical record reviews conducted by trained infection control professionals ensured that clinical judgment was applied when classifying cases.

## Conclusions

The CNISP surveillance data from this cohort study suggest that during waves 5 and 6 of the COVID-19 pandemic, Canadian hospitals experienced a surge in patients hospitalized with COVID-19, an increase in nosocomial transmission of COVID-19, and a decrease in severe outcomes. Rates of ICU admission and all-cause in-hospital death among patients hospitalized with COVID-19 were significantly higher among unvaccinated patients compared with those who were fully vaccinated or fully vaccinated with an additional dose. Despite a reduction in severe outcomes in waves 5 and 6, the burden of COVID-19 on Canadian hospitals was substantial. These data suggest the importance of COVID-19 vaccination in reducing both the burden on the Canadian health care system and severe outcomes associated with COVID-19.

## Notes

Supplement.
Data Sharing Statement
