# Error in Byline, Affiliations, and Contributions

**DOI:** 10.1001/jamanetworkopen.2023.17468

**Published:** 2023-05-23

In the Original Investigation titled “Trends in Severe Outcomes Among Adult and Pediatric Patients Hospitalized With COVID-19 in the Canadian Nosocomial Infection Surveillance Program, March 2020 to May 2022,”^1^ published April 20, 2023, Jessica Bartoszko’s surname was misspelled in the byline, author affiliations, and author contributions. This article has been corrected.
