# Error in Methods

**DOI:** 10.1001/jamanetworkopen.2023.29475

**Published:** 2023-08-08

In the Original Investigation titled “Trends in Severe Outcomes Among Adult and Pediatric Patients Hospitalized With COVID-19 in the Canadian Nosocomial Infection Surveillance Program, March 2020 to May 2022,”^1^ published April 20, 2023, the SARS-CoV-2 variant that was dominant in Canada during wave 4 of the COVID-19 pandemic (July 1 to December 25, 2021) was incorrectly identified as the Alpha variant instead of the Delta variant. This article has been corrected.

## References

[1] Mitchell R, Cayen J, Thampi N, . Trends in severe outcomes among adult and pediatric patients hospitalized with COVID-19 in the Canadian Nosocomial Infection Surveillance Program, March 2020 to May 2022. JAMA Netw Open. 2023;6(4):e239050. doi:10.1001/jamanetworkopen.2023.9050 37079304 PMC10119741

