# Supplementary material for: Trends in Severe Outcomes Among Adult and Pediatric Patients Hospitalized With COVID-19 in the Canadian Nosocomial Infection Surveillance Program, March 2020 to May 2022
Source: JAMA Netw Open. 2023 Apr 20;6(4):e239050. doi: 10.1001/jamanetworkopen.2023.9050 (PMC10119741; doi:10.1001/jamanetworkopen.2023.9050)
Supplement: Supplement. — Data Sharing Statement [file jamanetwopen-e239050-s001.pdf]

Data Sharing Statement

Mitchell. Trends in Severe Outcomes Among Adult and Pediatric Patients Hospitalized With COVID-19 in the Canadian Nosocomial Infection Surveillance Program, March 2020 to May 2022. *JAMA Netw Open*. Published April 20, 2023. doi:10.1001/jamanetworkopen.2023.9050

Data
Data available: Yes
Data types: Deidentified aggregate data, Data dictionary
How to access data: The study protocol is available. Data sharing requests will be considered and reviewed by the Public Health Agency of Canada and individual site investigators.
When available: With publication

Supporting Documents
Document types: None

Additional Information
Who can access the data: Researchers whose proposed use of the data has been approved.
Types of analyses: Data will be made available for the purpose of replication.
Mechanisms of data availability: Data will be made available after approval of a data sharing request and a signed data sharing agreement.
